# Detection of Vitiligo Through Machine Learning and Computer-Aided Techniques: A Systematic Review

**DOI:** 10.1155/bmri/3277546

**Published:** 2024-12-19

**Authors:** Sania Tanvir, Sidra Abid Syed, Samreen Hussain, Razia Zia, Munaf Rashid, Hira Zahid

**Affiliations:** ^1^Faculty of Electrical and Computer Engineering, Biomedical Engineering Department, Sir Syed University of Engineering and Technology, Karachi, Pakistan; ^2^Electronic Engineering Department, Faculty of Computer Systems Engineering, Electronic Engineering and Telecommunication Engineering, Dawood University of Engineering and Technology, Karachi, Pakistan; ^3^Faculty of Computer and Electrical Engineering, Electrical Engineering Department, Sir Syed University of Engineering and Technology, Karachi, Pakistan; ^4^Department of Software Engineering, Ziauddin University, Faculty of Engineering, Science, Technology and Management (ZUFESTM), Karachi, Pakistan; ^5^Department of Biomedical Engineering, Ziauddin University, Faculty of Engineering, Science, Technology and Management (ZUFESTM), Karachi, Pakistan

**Keywords:** detection, diagnosis, image segmentation, machine learning, skin disease, systematic review, vitiligo

## Abstract

**Background and Objective:** Vitiligo is a chronic skin damage disease, triggered by differential melanocyte death. Vitiligo (0.5%–1% of the population) is one of the most severe skin conditions. In general, the foundation of the condition of vitiligo remains gradual patchy loss of skin pigmentation, overlying blood, and sometimes mucus. This paper provides a systematic review of the relevant publications and conference papers based on the subject of vitiligo diagnosis and confirmation through computer-aided machine learning (ML) techniques.

**Materials and Methods:** A search was conducted using a predetermined set of keywords across three databases, namely, Science Direct, PubMed, and IEEE Xplore. The selection process involved the application of eligibility criteria, which led to the inclusion of research published in reputable journals and conference proceedings up until June 2024. These selected papers were then subjected to full-text screening for additional analysis. Research publications that involved application of ML techniques with targeted population of vitiligo were selected for further systematic review.

**Results:** Ten selected and screened studies are included in this systematic review after applying eligibility criteria along with inclusion and exclusion criteria applied on initial search result which was 244 studies based on vitiligo. Priority is given to those studies only which use ML techniques to perform detection and diagnosis on vitiligo-targeted population. Data analysis was carried out only from the selected and screened research articles that were published in authentic journals and conference proceedings.

**Conclusion:** The importance of applying ML techniques in the clinical diagnosis of vitiligo can give more accurate results and at the same also eliminate the need of biased human judgement. Based on a comprehensive examination of the research, encompassing the methodologies employed and the metrics utilized to assess outcomes, it was determined that there is a need for further research and investigation regarding the application of ML algorithm for the detection and diagnosis of vitiligo with different datasets and more feature extraction.

## 1. Introduction

Vitiligo is a chronic skin damage disease, triggered by differential melanocyte death. Vitiligo (0.5%–1% of the population) is one of the most severe skin conditions. It has a strong stigmatic impact, but a near-orphan medical status [1]. Typical vitiligo lesions can be described as milky white, nonscaly, slightly separate macules. According to a recent international consensus conference, vitiligo can be divided into two main forms—that is, vitiligo that is segmental and nonsegmental vitiligo. The most severe type of this debilitating condition is nonsegmental vitiligo, characterized by symmetrical white and bilateral spots. Many clinical subtypes including generic, acrofacial and universal types have been identified, all of which are bilateral in nature. Segmental vitiligo is less common than nonsegmental vitiligo and is normally distributed separately. In general, the foundation of the condition of vitiligo remains gradual patchy loss of skin pigmentation, overlying blood, and sometimes mucus [2]. Our knowledge of disease pathogenesis, which now contributes to new treatment strategies focused on targeted therapy, has substantially improved recent basic and translation work [3, 4].

Topical steroids and calcineurin inhibitors that suppress immune responses in the surface skin of an inflammatory infiltrating agent are among the existing treatments in vitiligo. These are most useful in the treatment of localized disease, with a surface area of less than 5% (BSA). For more common diseases, or diseases that evolve and are infectious with the appearance of new lesions, phototherapy is initially prescribed as psoralen plus UVA (PUVA), but now predominantly as narrowband UVB (nbUVB). Excimer lasers that produce a single wavelength of light in the nbUVB range are also effective in treating or desiring a faster response localized diseases that are not immune to topical therapies [5]. Vitiligo, which is highly active, can be controlled by oral pulse steroid therapy, which persists during treatment (usually nbUVB). Current treatments for vitiligo though moderately effective are both financially and technologically onerous [4].

In 2012, the findings of a structural evaluation were outlined and objectively understood, evaluating some significance structures in patients with all forms of vitiligo, the measuring properties of the clinician, patient, and observer performance [6]. Various measurement instruments such as the European Task Force (ETF) test for vitiligo have been tested [1]. On calculating error, the VETF score was assessed. No interpretability details were found, since there were no records of small significant improvements or slight significant differences. The above has also tested a rather well-known Vitiligo Rating Index (VASI) [4]. The only assessment property calculated in the VASI is the criterion validity. Evidence for the validity of the study became unclear because of the poor quality of the tools used. There was little evidence of positive efficacy of the point counting method in point counting [5] because a correlation coefficient of equal methodological quality greater than 0.70 is demonstrated.

For three reasons, current evidence of measurement properties of vitiligo outcome measures is insufficient. First, there was a very small number of items used. Secondly, the majority of experiments analyzed was methodologically inadequate. Thirdly, there is no clear clinical significance. Recommendation should be developed with care because of this lack of evidence on the use of specific measuring instruments [6]. Due to lack and limited number of studies with invalid interpretations, there is a need of computer-aided techniques to perform feature extraction and diagnosis of many skin diseases and in this case vitiligo. These computer-aided techniques can eliminate human judgement in case of skin disease to an extent. One of the most commonly observed diseases in humans is skin conditions. Skin disorders cause a lot of trouble in individuals with disfigurement and related difficulties [1, 7].

Therefore, it is difficult to diagnose skin disease. A number of visual signs such as lesion shape, body size, texture, volume, and structure of lesions can be used to diagnose a skin disease. The recognition process can be quite difficult if the individual components are analyzed separately [8, 9]. A computer-aided diagnostic method is more accurate and effective, as opposed to a test by human experts, which is more dependent on the subjective evaluation, and hard to replicate. The current state-of-the-art computerized diagnostic systems [10] can achieve very good performance in certain skin cancers, such as melanoma, by integrating them with some popular classification tools (such as SVM and artificial neural network (ANN)). The separation of the human features is not suited to a system of classification for systemic skin disease. On the one side, hand-built features are usually used for one or more specific skin diseases. It is impossible to use for other types or datasets. On the other hand, human invention for every skin disease is impossible, given the diversity of skin diseases [7]. One way to fix this problem is to use functional learning that reduces practical engineering needs and lets the machine determine which functionality to use. Several classification systems based on learning functions have been introduced in recent years [11, 12]. Deep neural networks (convolutional neural network (CNN)) are becoming very popular in recent years in the classification of features and artifacts. The use of high-performance GPU requires the network to be built on a large dataset in order to improve performance [7].

This study presents a comprehensive analysis of the pertinent literature and conference proceedings pertaining to the topic of computer-assisted strategies for diagnosing and confirming vitiligo. The objective of this review is to examine, synthesize, evaluate, and debate a collection of research publications in order to assess their specificities, outcomes, and reliability. Our investigation is centered on the examination of highly cited scholarly articles from reputable library databases, including PubMed, IEEE Xplore, and Science Direct, up to June 2020.

The primary objective of this study is to evaluate the present effectiveness of several machine learning (ML) techniques employed for the diagnosis of vitiligo. Additionally, it intends to investigate the advancements, limitations, and challenges encountered in this field, while also identifying areas that require further investigation. This is the first systematic review of ML techniques for the detection of vitiligo, which extensively reviews all related research articles from major journals and conferences in relation to specific established research questions to the best of our knowledge. The important contribution of this paper is to perform a systematic review on the detection of vitiligo through ML techniques and identify the gap for research in this field.

## 2. Materials and Method

Over the past decade, there has been a notable rise in the quantity of research papers produced in the field of biomedical literature, with a specific emphasis on tropical medicine and health. However, it should be noted that the existing studies exhibit significant heterogeneity in terms of their operational efficiency, subject matter, and their potential impact on the investigative problem. This heterogeneity further complicates the process of establishing evidence and reaching consensus on the findings [13]. The methodology of this paper has been adopted by Syed, Rashid, and Hussain [14].

The evidence-based pyramid sets a rigorous level of proof, which is achieved by the systematic examination and meta-analysis of studies (SR/MAs). A well-executed systematic review/meta-analysis is therefore seen as a feasible strategy to ensure that healthcare professionals remain informed about contemporary evidence-based medicine. Furthermore, despite the provision of enhanced instructions for the effective execution of a systematic review, our investigation reveals that the fundamental stages persist in commencing with the task of formulating the research question, identifying pertinent studies through the establishment of inclusion and exclusion criteria, conducting a comprehensive search for relevant articles, assessing the methodological quality of the selected studies, synthesizing the gathered data, and interpreting the resulting findings. Researchers possess the ability to address a wide range of problems, even in the absence of specific information [15]. This review adhered to the principles outlined in the Preferred Reporting Items for Systematic Reviews and Meta-Analyses (PRISMA) in terms of its planning, execution, and reporting.

### 2.1. Research Question

To define the work limits, the RQs were introduced. The requirements (PICO) [2, 14] have been designed to consider four types of study questions. This study is also drawn on the base of PICO format which form the research question as follows:

• P (population) = patients that have vitiligo skin disease.
• I (intervention) = detection with data given in the form of images
• C (comparison) = ML algorithms and skin segmentation techniques
• O (outcome) = report accuracies and compare them.

### 2.2. Search Keyword Strategy

A collection of search queries was created using the Boolean operator to combine appropriate synonyms and alternate terms: the operation AND narrows down and confines the search, while the operator OR broadens and amplifies it [14, 16]. So with help of these Boolean operators, the search term was formulated as follows: (vitiligo) AND (“computer vision” OR “neural network” OR “artificial intelligence” OR “pattern recognition” OR “machine learning”).

### 2.3. Databases and Information Sources

**Figure 1:**
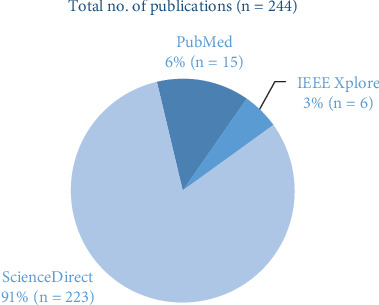
Pie chart of no. of publications in the initial stage before applying any sort of criteria.

A systematic search was conducted in three prominent databases, namely, PubMed, IEEE Xplore, and Science Direct, to identify peer-reviewed papers. The search parameters in ScienceDirect were limited to include review articles, research papers, conference abstracts, correspondences, data articles, debates, and case reports. The search of all three databases has been conducted up until June 2024. The formulated keywords in Section 2.2 have been used to perform search in these databases. The search results were in PubMed (*n* = 15), IEEE Xplore (*n* = 6), and ScienceDirect (*n* = 223). So the total number of search results was *n* = 244 initially (Figure 1).

The EndNote online system was utilized to record and organize search results, and subsequently, a table of data was collected from each selected document. The entire texts of articles that were considered possibly suitable for registration were put into the EndNote Internet platform, provided by Clarivate Analytics. The initial search involved utilizing the designated search phrases for each chosen database, encompassing comprehensive documents from both scholarly publications and conference proceedings. The approach has yielded a substantial number of finds that lack relevance, prompting a decision to restrict the search to the document's title and content type. The determination of further research is contingent upon referencing the sources of the relevant studies that have been identified. Following the compilation of primary research papers, a thorough examination was conducted on the titles and abstracts of these studies in order to identify those that were pertinent to the research at hand. A comprehensive examination has been conducted to evaluate the pertinent research in an ongoing inquiry.

### 2.4. Eligibility Criteria

• This study primarily examined peer-reviewed studies that employed ML techniques for the purpose of identifying white patches in photographs of vitiligo. Additionally, three criteria were employed for the purpose of screening and evaluating publications:
• Comparative skin photos of patients with vitiligo versus patients without vitiligo. Our primary focus was on reviewing research articles that are relevant to the first criterion, in order to gain a comprehensive understanding of the problem from the perspectives of ML and implementation. This compilation solely comprises papers that have employed skin photographs as a means to cure the vitiligo illness.
• The application of ML has proven effective in accurately distinguishing between individuals with healthy skin and those affected by vitiligo. The second criteria pertain to the imperative of ensuring that the research articles picked employ methodologies grounded in the principles and techniques of ML. This criterion excludes studies that do not include ML or an algorithm for illness definition. Additionally, this criterion excludes articles that are exclusively focused on qualitative analysis.
• The accuracy and quantitative analyses are documented in writing. The third criteria highlight the inclusion of image identification software for illness in the selected research publications. This examination demonstrates the precision of ML and the efficacy of the methodologies employed in the chosen papers for analysis, which were subjected to quantitative scrutiny and verification.

The use of inclusion and exclusion criteria was employed to identify and report research publications that were deemed irrelevant. This examination paper provides an overview of the inclusion and exclusion criteria that were employed.

#### 2.4.1. Inclusion Criteria

• The objective of this study is to explore research publications that utilize skin pictures as a dataset for the purpose of illness prediction.
• This research paper focuses on the use of ML techniques.
• The articles encompass various image segmentation approaches, applications, and software that were aimed at detecting diseases using picture analysis.
• All articles are written in the English language.
• A published narrative is often included in either a journal or a conference proceeding.

#### 2.4.2. Exclusion Criteria

• Research articles that were not focusing on skin images as a data to detect the disease.
• Research articles that do not focus on ML or any software for detection.
• Conduct a study on research that has not been documented in the English language.
• Research studies that have not been published in any academic journal or presented at any conference proceedings.

**Figure 2:**
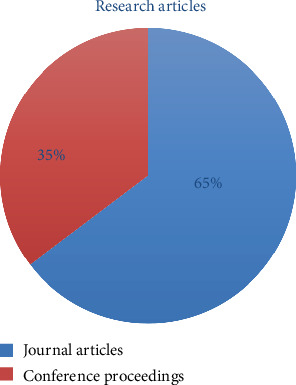
Pie chart of no. of publications included after screening and after applying exclusion and inclusion criteria.

After applying this eligibility criteria along with both inclusion and exclusion criteria, there were a total of ten studies (*n* = 17) out of which five were journal publications (*n* = 11) and five were conference publications (*n* = 6) in Figure 2 that are used for further evaluation in this review paper.

For each of the outcomes, a customized method of data collection was created, that is, disease and ML. Data analysis was carried out only from the published research articles that were from genuine journals and conferences. That analysis has extracted the characteristics are in Table 1, including study details (author name, publication year, and journal/conference), datasets (no. of images, origin, and open access/close access), ML and technique, and outcome measurements (accuracy/*p* value/Jaccard index (JI), specificity, and sensitivity).

### 2.5. Assessment of Risk of Bias

After mutual understanding between the authors, the risk of bias of each screened and selected research publication included in this systematic review was calculated. This was executed in order to assess the validity of each selected research article. A common tool called ROBIS and suggested by Whiting et al. was used to evaluate the risk of bias [33]. The writers discussed and agreed upon certain criteria, thus assessing the risk of bias. The results of the randomization process, deviations from intended interventions, distortions caused by missing results, measurement of the results, and bias in the selection of the reported result included the biases. Based on these five parameters, each analysis was analyzed, which measured high, low, and no details. There were some questions. Studies that contained a detailed and adequate methodology and therefore had a low chance of presenting a potential risk of bias were considered to be at little risk of bias for a criterion. Research has also been found to encapsulate a high risk of distortion, because the research paper includes an insufficient technique to eliminate potential distortion. Bias due to randomization process entails to any biasness emerging from using a technique or algorithm randomly. Another parameter taken into consideration is the biasness arising due to inappropriate interventions. This relates to executing an intervention that was not initially intended and thus creating deviations. Bias can also arise in a study if certain data is missed out or if outcome data is inappropriately measured. Apart from this, biasness can also result if certain results are considered only thus leaving the rest out.

## 3. Results

This report summarizes the results of the primary studies chosen and outlines the conclusions, the publishing years and origins, and the predictive evaluation results. The results of the study are summarized below.

### 3.1. Identification of Studies

**Figure 3:**
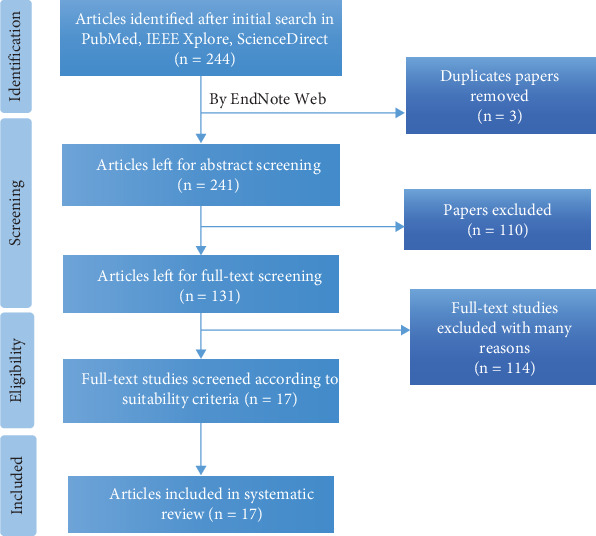
PRISMA flowchart according to PRISMA guidelines and statement recommendations.

The search for literature review resulted in 244 publications, out of which we excluded three duplicate publications (Figure 3). Some articles were also removed due to language barriers. One hundred and ten reviews were then removed after reading the titles and abstracts, and 131 publications were left for further study. After examining methods, 121 publications were retained for further inspection and after thorough review of papers, 17 publications satisfied the criteria which are summarized in Table 1 and were included for the study of systematic review. Table 1 summarizes the publications in terms of their objectives, methods, algorithms used, analysis, accuracy, results, and conclusion investigated.

### 3.2. Study Characteristics

The studies that are summarized in Table 1 can be outlined in three sections for the systematic review for quantitative analysis. The first in Section 3.4 would be the use of supervised and unsupervised ML techniques used in the detection of vitiligo and the results of these techniques in the *p* value, accuracy, or JI value. These studies were aimed at how these techniques are effective in the diagnosis that is further used to treat the disease accordingly because vitiligo has many types and every type has a different type of treatment and level or depigmentation [2]. In this review, only six research studies report the no. of patients and out of them, only two report age, and the reported age is above 15 and varies from 18 to 55 years (Table 1). This systematic review talks about the outcome of applied ML techniques and their effectiveness that could be applied for clinical trials.

### 3.3. Publication Years

**Figure 4:**
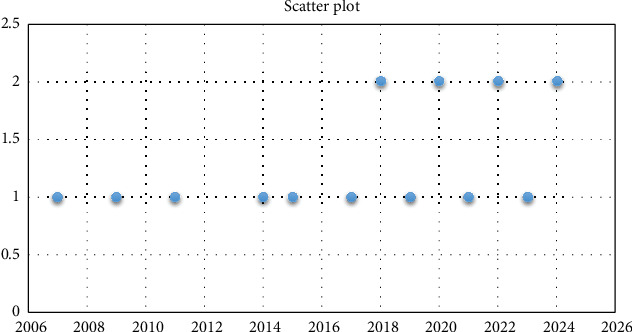
Scatter plot of publication year of selected and screened studies.

From Table 1 in Section 3.4, we can observe that all the selected and screened stories are in between 2005 and 2020. Hence, Figure 4 shows that most of the work on vitiligo has been done in the recent decade.

### 3.4. Quantitative Analysis

#### 3.4.1. Key Observations From Table 1

1. Techniques used: The paper highlights a wide variety of ML and deep learning (DL) techniques employed in vitiligo detection and classification. A significant portion of the reviewed studies utilized supervised learning algorithms. Among these, CNNs, along with more advanced architectures like ResNet and Swin Transformer, stood out for their exceptional performance. CNN-based models were widely preferred due to their superior ability to learn hierarchical features and patterns in medical images. Other methods, such as ANNs and Naive Bayes, were also explored but often demonstrated lower performance compared to CNN-based techniques. Additionally, unsupervised learning methods like *k*-means clustering and agglomerative clustering were implemented in earlier studies. However, these techniques generally exhibited lower accuracy, showcasing their limitations in handling the complexity of skin lesion detection compared to newer methods. This shift from traditional ML to DL highlights the advancements in computational power and the availability of larger datasets, which have allowed more complex models to flourish in this domain.
2. Accuracy and performance: Accuracy has been a critical performance metric in the studies reviewed, and DL models, particularly CNNs and their variants, consistently outperformed other algorithms. For example, studies like Zhang et al. [24] and Sharma et al. [23] achieved accuracy rates of 99%, setting a high benchmark in vitiligo classification. These models benefitted from advanced image processing capabilities and large, annotated datasets, allowing them to detect intricate patterns and features in the skin images with minimal human intervention. On the other hand, traditional techniques, such as *k*-means clustering and KL divergence–based clustering, which were used in earlier studies, reported significantly lower accuracy scores. For instance, Kislal and Halasz [19] reported only 59% accuracy, which reflects the limitations of earlier unsupervised learning techniques that were unable to fully capture the nuances of skin pigmentation differences. The gradual increase in accuracy over time indicates that newer DL architectures not only improve diagnostic accuracy but also reduce the time and resources required for manual feature extraction.
3. Sensitivity and specificity: Sensitivity and specificity are crucial metrics in medical diagnosis, as they indicate a model's ability to correctly identify true positives and avoid false positives, respectively. Across the reviewed studies, the sensitivity values varied widely, ranging from 47% to 100%, depending on the model and dataset used. This variation in sensitivity highlights the challenges in consistently detecting all cases of vitiligo, particularly in patients with mild or early-stage symptoms. For instance, models like those of Sharma et al. [23] demonstrated 100% sensitivity, meaning that all positive cases were accurately identified. This is a significant achievement for vitiligo diagnosis, as missing cases could lead to delayed treatment. Specificity, though reported in fewer studies, also showcased the efficiency of certain models in avoiding false positives. The specificity rates reached as high as 99% in the same study by Sharma et al., underscoring the effectiveness of DL models in balancing sensitivity and specificity. However, older models such as those used by Gupta et al. [31] and Kislal and Halasz [19] were prone to false positives, achieving lower specificity values, which suggests that these models may have mistakenly identified nonvitiligo lesions as vitiligo, leading to overdiagnosis. This imbalance in sensitivity and specificity remains a challenge in ML applications for medical diagnostics
4. Dataset sources: The diversity and size of datasets used in these studies play a significant role in determining the models' performance. A wide range of data sources were employed, including proprietary clinical datasets, publicly available datasets like DermNet, and large Internet-based collections from Google Images and Kaggle. The dataset sizes varied significantly, from as few as 31 images used by Aydin et al. [5] to over 4000 images in studies like Zhong et al. [21]. Larger datasets generally resulted in more robust and generalizable models, as they provided a broader range of examples for training. For instance, studies utilizing large-scale datasets with over 4000 images consistently achieved high accuracies, as seen in the Zhong et al.'s (2024) study, where the dataset size contributed to a 99% accuracy rate. Smaller datasets, on the other hand, limited the models' ability to generalize to new data. This was evident in the study by Aydin et al. [5], where a limited dataset of only 31 images resulted in lower model performance. The availability of diverse and large datasets has allowed DL models to thrive, enabling more accurate and reliable vitiligo detection. The paper emphasizes the need for larger and more diverse datasets in future research to further improve the generalizability and robustness of these models.
5. Journal vs. conference publications: The paper draws a distinction between journal and conference publications, noting that journal articles tend to report more mature and thoroughly tested methods, often with higher accuracy rates compared to conference papers. For example, journal publications like Guo et al. [25] report an accuracy of 92.91%, showcasing a well-tested CNN-based model trained on a substantial dataset. In contrast, some conference papers, such as Gupta et al. [31], which focused on agglomerative clustering, reported lower accuracy scores, often due to the use of older or less advanced techniques. This trend suggests that journal publications, which typically undergo more rigorous peer review, may present more reliable and refined models. Conference papers, while often introducing novel ideas or preliminary findings, may lack the extensive testing and optimization found in journal studies. Therefore, the publication medium serves as a useful indicator of the model's development stage and reliability. This observation aligns with the general trend in scientific research, where journal articles are often seen as more authoritative sources, providing deeper insights and more conclusive findings.

**Figure 5:**
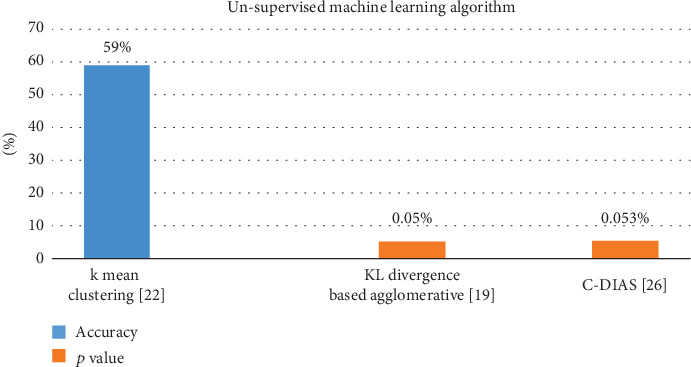
Comparison of unsupervised techniques outcomes.

The first quantitative analysis in this systematic review has been conducted between all the unsupervised techniques algorithm used in studies [19, 20, 28] showed in Figure 5. Unsupervised use the dataset type to learn. The neuron has no training data to learn the difference between all the neural network features separated by the area it simply knows as a cluster. The name of this type of learning is unsupervised [27]. Kislal and Halasz in [19] uses *k*-means clustering technique and predicted result in form of repigmentation accuracy, which is 59%, and it is considered very low in terms of final outcome. Our web-based software allows doctors to track and quantify the effects of therapies in terms of the extent of these diseases. There reported repigmentation value does not seem authentic because they use some manual method to compare their outcome with which is not valuable in terms of ML. Shamsudin et al. in [20] used the C-DIAS method and report their outcome in the form of *p* value, and their reported *p* value is 0.053 which is high as compared to the *p* value reported in Das Gupta et al. [28] as < 0.05. Das Gupta et al. used KL divergence–based agglomerative clustering. This article presents a symmetric KL divergence based on a multilevel segment agglomerative clustering system in vitiligo frames. They propose symmetric Kullback–Leibler (KL) divergence as the optimal distance parameter in between the two very generally divided clusters. Based on the clustering, hierarchical in specific, routine of image segmentation has been formed. The super pixels provide a simple fundamental that can be used to quantify local image value. They use to reduce the complex specifications of selected task of image processing and are increasingly useful in the segmentation of images. They provide a comparative analysis of established techniques and prove that both performance and runtime of the method that has been proposed overtake or do not match with most of the already existing techniques. For this form, the Spearman ranking association is 0.9998 and for Expert 2 is 0.9636 with *p* value < 0.05. Please note that the value of correlation in between the two experts is also 0.9636, indicating the reproductiveness of the measurement technique for the disease-based area, whereas Shamsudin et al. in [20] objectively assess the vitiligo reaction of the patient with the use of C-DIAS and compared with PGA. As assessed by the digital method (MPR-C-DIAS) or PGA, the primary effectiveness end point was the average depigment rate (MPR). The answer was graded as none (0%), fair (1 to 25%), reasonable (26 to 50%), strong (51 to 55%), and outstanding (76 to 100%). Out of 56 individuals, 44 (79%) responded. MPR-C-DIAS. In general, the response was reasonable in 22 patients (39%), mild in 21 patients (40%), and strong in one patient (2%). A total of 39 patients (70%) responded with PGA. In 27 (48%), 10 (18%), and two (4%) patients, repigmentation was mildest, good, or excellent. The consistency test was 0.17 (*p* = 0.053) and suggested that there was weak agreement between the two methods of evaluation, but this was not statistically significant. In addition to treatment, C-DIAS can be used for the purposeful analysis of vitiligo skin lesions for repigmentation and depigmentation.

**Figure 6:**
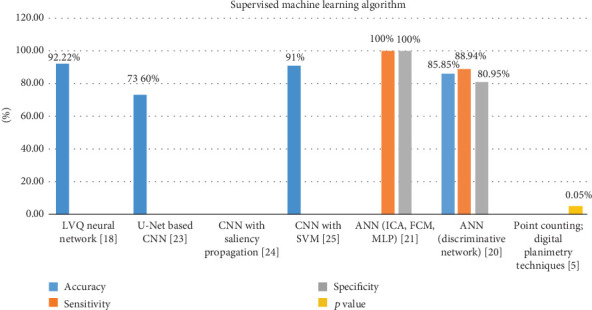
Comparison of supervised technique outcomes.

The second quantitative analysis in this systematic review has been conducted on all the studies which used supervised technique algorithm. It is clearly visible in Figure 6 that out of all supervised studies, only Cazzaniga et al. in [17] have reported all the outcomes that are required and considered proper outcome in ML. Cazzaniga et al. reported that accuracy is 85.85% with sensitivity value which is 89% and specificity value which is 80%. The ML technique used by Cazzaniga et al. in [17] is ANN along with discriminative and regression network. Digital image study of UVB-reflected images assessed the level of repigmentation. As no strong relationship was initially reported between any specific predictive parameter and outcome, ANNs are used. The results have been divided into two groups of respondents and nonrespondents using a period-response optimal threshold model. The preparation of a discriminatory network to classify respondents against nonrespondents was carried out. A regression network was then used for contestants to measure repigmentation time, whereas ANN has also been used by Chica et al. in [18] along with independent component analysis (ICA), fuzzy *C*-mean (FCM), and multilayer perceptron (MLP). Chica et al. did not report accuracy but they did report sensitivity and specificity value. For ANN with ICA, their sensitivity value varies from 96% to 100% in all 20 tests and specificity value varies from 97% to 100% in all 20 tests. For ANN with FCM, their sensitivity value varies from 99% to 100% in all 20 tests and specificity value varies from 47% to 100% in all 20 tests. For ANN with MLP, their sensitivity value varies from 98% to 100% in all 20 tests and specificity value varies from 98% to 100% in all 20 tests. Reporting method is very good and detailed in the study of Chica et al. [18]. The authors provide an innovative approach utilizing ANNs to discern regions of healthy skin and skin affected by vitiligo, leveraging the properties of light-skin interaction. Photographs depicting distinct regions of skin affected by vitiligo were employed. The independent variables used for this study are the categorization of skin type, the quantification of skin affected by vitiligo, and the quantification of depigmented skin. Given the aforementioned variables, the studies were systematically arranged in an orthogonal table. The authors conducted an analysis of the outcomes obtained from the methodology, considering three key parameters: sensitivity, specificity, and F1-score. Subsequently, a comparison was made between the findings of this methodology and those of other techniques provided in related studies. The findings indicate that the approach provided possesses the capacity to be employed in clinical settings. It is worth mentioning that enhancing the training patterns has the potential to yield substantial improvements in performance.

CNNs, a family of algorithms with significant impact in diverse computer vision applications, have garnered considerable attention across several fields, including the field of radiology. CNNs are structured to acquire spatial hierarchies by using various components such as pooling layers, convolutional layers, and fully connected layers, together with mechanisms for automated and adaptive context propagation. The user has provided a numerical reference without any accompanying text or context. CNN is a widely employed DL technique utilized for the resolution of complex issues [34]. CNN is a computational model that falls within the category of DL techniques. This approach effectively addresses the constraints associated with conventional machines [35]. In our selected and screened studies, there in are three studies [29–31] in which CNN is used as a ML technique. In Figure 6, we can see that only Low, Huang, and Raina in [29] and Gupta et al. in [31] have reported accuracy in number and Bian et al. in [30] have reported accuracy in words. Low, Huang, and Raina in [29] used U-Net-based CNN. They establish a CNN that performs such segmentations quickly and robustly without manual intervention. They use the U-Net to create the first segmentation of the lesion with a modified contracting path. Then, you use high-confidence pixels as “seeds” to segment the watershed algorithm. The network consists of 247 images of various sizes, complexity, and anatomy. Overpassing the state-of-the-art U-Net, our network scores 73.6% of the JI (comparison to 36.7%). Segmentation takes place in few seconds, a considerable improvement compared with the semiautonomous solution previously proposed. Gupta et al. in [31] have proposed a framework to identify the different types of skin injuries caused by diabetes. The system architecture utilizes enhanced photos to identify regions of skin injury. In order to achieve local and international characteristics, visual saliency and the CNN model are used for extraction. The results indicate explicitly that the architecture suggested can be effectively used to identify different categories of skin damage. This can therefore be used to assist humans with diabetes-related skin injuries in their everyday lives. Bian et al. in [30] have reported accuracy in words as “After considerable trials, we find the algorithm is robust.” In conclusion of their study, they wrote “We provided competitive evaluation against existing methods and showed the proposed method outperformed others”. Bian et al. in [30] trained a CNN classifier. We train from scratch a CNN model to categorize the images into the Vit2019 data package simply by placing a global average pooling (GAP) on the convolution mapping a fully connected layer just before. Then, the activation by-product of CNN was used to locate roughly the vitiligo region. The classifier's accuracy can to some degree reflect the quality of the activation map. To train the network of classification, Vit2019 is arbitrarily divided into training = 70% and testing = 30%; then, the ResNet-50 network is misclassified with 4.27% of the test samples.

### 3.5. Risk of Bias Within Studies

**Figure 7:**
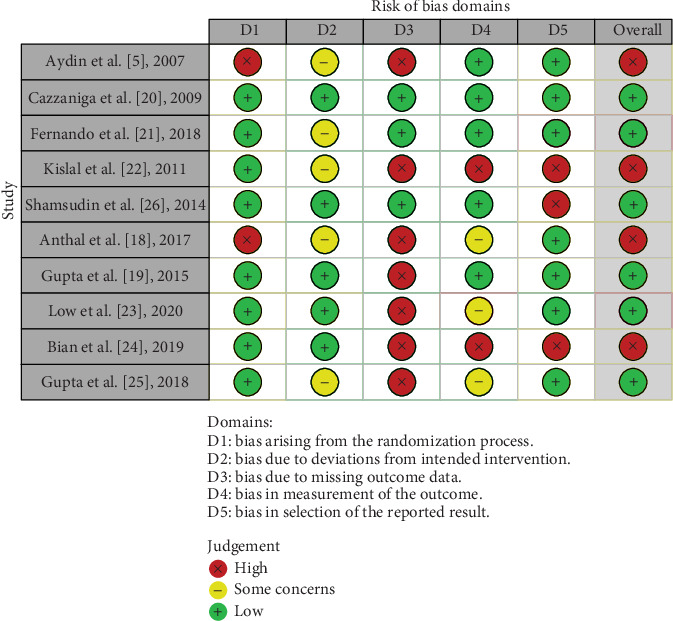
Traffic light plot illustrating risk of bias.

**Figure 8:**
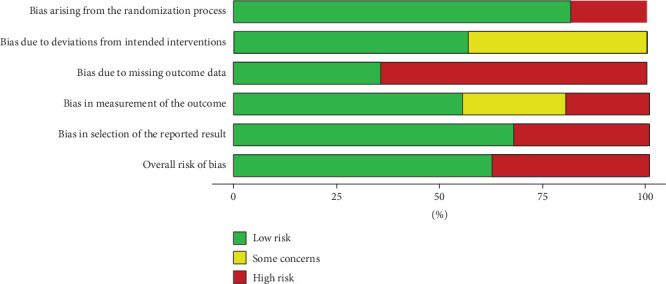
Weight plot illustrating risk of bias.

The judgements and assessments pertaining to each risk of bias domain are exhibited in the form of a traffic light plotted in Figure 7. Apart from this, for the judgements pertaining to low risk (green), some concerns (yellow), and high risks (red), they have also been illustrated in the form of a weighted bar plot in Figure 8. Two out of ten selected articles had a very low risk of the bias arising because of any randomization process. Three out of ten studies have low risk in the D3 bias and the rest of them are at high risk of bias. Overall, four studies are at high risk and six are at low risk.

## 4. Discussion

All the screened and selected 10 research publications that are included in this systematic review after going through an eligibility criteria along with inclusion and exclusion criteria comprised of assessments being executed on the diagnosis of vitiligo. These research studies demonstrated that ML techniques and algorithms in comparison to other traditional and conventional methods are more effective in analyzing and detecting vitiligo. According to the primary objective for which this systematic review was executed and relevant inquiries and investigations, it can be suggested that machine and DL techniques such as CNN and ANN are more efficient. In 2012, the findings of a structural evaluation were outlined and objectively understood, evaluating some significance structures in patients with all forms of vitiligo, the measuring properties of the clinician, patient, and observer performance [6]. Various measurement instruments such as the ETF test for vitiligo have been tested [1]. On calculating error, the VETF score was assessed. No interpretability details were found, since there were no records of small significant improvements or slight significant differences. The above has also tested a rather well-known VASI [4]. The current state-of-the-art computerized diagnostic systems [10] can achieve very good performance in certain skin cancers, such as melanoma, by integrating them with some popular classification tools (such as CNN and ANN).

Out of all the studies, Cazzaniga et al. in [17] have reported all the three things, that is, accuracy, sensitivity, and specificity, that are the requirement of good ML reporting. We can analyze that from Table 1 that most of the papers fail to reports all the outcome measurements that are considered desirable in ML. Aydin et al. in 2007 [5] use a technique that is not a ML technique but depicted a good result. In 2007, surface areas of 31 vitiligo lesions were calculated by five volunteers using point counting and optical planimetry. Twice with an interval of 2 weeks, three independent observers checked the areas mentioned with the point counting method. Any single observer using digital planimetry has calculated the same lesions. An inter- and intraobserver interaction test was used to compare the estimation results of three observers. For all measurements, there was significant inter- and intraobserver consensus. Every observer's observations of the point counting and the digital planimetry tool also had a major similarity. There were no major differences in surface results obtained by the two methods (*p* > 0.05). The dot counting method can be used to measure the area of the vitiligo as a simple and reliable method.

Similarly, Anthal et al. in 2017 [27] used LVQ neural network which also seems very promising in terms of accuracy because its reporting accuracy is 92%. The vector quantification of learning is a type of neuronal network that uses a clustering and classification combination. The LVQ neural network involves three layers: input, hidden, and output; clusters between input and hidden layers and classifications are extended from the secret to output layer. In this case, a neural network with learning vector quantization classifies a vitiligo image as an infection in the affected region vs. the uninfluenced environment. The LVQ neural network is remarkably well implemented with a 92.22% exactness and 0.810 kappa value which is remarkable for the approach being proposed.

### 4.1. Limitations

Some of the studies also reported limitations, for example, in [17], where the presence of numerous equilibrium predictor subsets led to multiple solutions. A broader collection of vitiligo lesion characteristics and treatments would enhance the development of a more predictive model of excimer laser treatment. Asset constraints were a maximum in [29], limiting the model training time. We would have benefited from additional resources, including computational power, to further optimize the models.

Furthermore, the potential of generative adversarial networks (GANs) to improve model performance by generating synthetic samples could address the issue of limited data in vitiligo research. GANs could be applied to create realistic synthetic images of vitiligo lesions, which could augment the dataset, thus mitigating the small sample size. The key benefit of GANs is their ability to create high-fidelity, realistic data that can help in training ML models more effectively, especially in situations where real-world data is scarce. By generating synthetic lesion images with varying characteristics (e.g., size, shape, and color of depigmented areas), GANs can help diversify the training set, potentially leading to more robust and generalizable models.

However, while GANs can produce valuable synthetic data, there are challenges and limitations associated with their use. Training GANs can be computationally expensive and time-consuming, requiring substantial resources to produce high-quality results. Additionally, GANs may sometimes generate unrealistic or low-quality samples, which can negatively impact the model's performance if not carefully validated. Moreover, there is a risk of overfitting to synthetic data if the generated samples do not accurately reflect real-world variability. Thus, while GANs offer a promising approach to addressing the challenge of limited data in vitiligo research, careful consideration must be given to their limitations and proper validation of the generated data is essential.

When considering the limitations of this systematic review, it is important to acknowledge the small number of papers included in the analysis. Furthermore, the inclusion criteria for this study encompassed only papers published in the English language, which may limit the representation of research conducted in non–English-speaking countries and thus affect the generalizability of the findings. Lastly, the search method employed in this study may have inadvertently overlooked several pertinent research articles, particularly those published in lesser-known journals or conferences, which were excluded from the systematic review.

## 5. Conclusion

Vitiligo is a chronic skin damage disease, triggered by differential melanocyte death. Vitiligo (0.5%–1% of the population) is one of the most severe skin conditions. It has a strong stigmatic impact, but a near-orphan medical status. Our knowledge of disease pathogenesis, which now contributes to new treatment strategies focused on targeted therapy, has substantially improved recent basic and translation work. For three reasons, current evidence of measurement properties of vitiligo outcome measures is insufficient. Second, there were a very small number of items used. Secondly, the majority of experiments analyzed was methodologically inadequate. Thirdly, there is no clear clinical significance. Recommendation should be developed with care because of this lack of evidence on the use of specific measuring instruments. Due to lack and limited number of studies with invalid interpretations, there is a need of computer-aided techniques to perform feature extraction and diagnosis of many skin disease and in this case vitiligo. These computer-aided techniques can eliminate human judgement in case of skin disease to an extent. The important contribution of this paper is that it reviews outcomes and accuracy of 10 relevant articles and also identifies the gap for research in this field. Six out of total 10 studies pertain to low-risk bias and four research article show high-risk bias due to due to unavailability of outcome measurements. After detailed analysis of the studies including the techniques used and outcome measurements, it was concluded that there is a need for further research and investigation regarding the application of ML algorithm for the detection and diagnosis of vitiligo with different datasets and more feature extraction. Furthermore, there is also a need of more authentic and open access datasets for experiments with ethical considerations.

## Figures and Tables

**Table 1:** Summary of the 10 screened and selected studies.

**S. No**	**Author and year of publication**	**Objective**	**ML algorithm/technique**	**Dataset source**	**Dataset size**	**Accuracy/** ***p* value/JI**	**Sensitivity**	**Specificity**
Journal								
1	Aydin et al. [5], 2007	Assess the accuracy and reliability of the system of point counting and equate it with modern planimetry technology for vitiligo surface estimation	Point counting; digital planimetry techniques (supervised)	Vitiligo lesions of five volunteers	*n* = 31	*p* value < 0.05	NA	NA
2	Cazzaniga et al. [17], 2009	Build a statistical rule based on randomized excimer laser trial data in vitiligo	ANN (discriminative and regression network) (supervised)	120 patients	*n* = 325	Ac = 85.85%	88.94%	80.95%
3	Chica et al. [18], 2018	To propose a novel method based on ANN that uses characteristics of the interaction of light with the skin to determine areas of healthy skin and skin with vitiligo	ANN (ICA, FCM, and MLP) (supervised)	Patients (18 and 55 years)	NA	NA	Vary between 96% and 100% in 20 tests for all three methods	Vary between 47% and 100% in 20 tests for all three methods
4	Kislal and Halasz [19], 2011	To propose a new semiautomated system to evaluate the extent of digital photographic vitiligo which is quick, simple, reliable, and objective	*k*-means clustering (unsupervised)	NA	NA	Ac = 59%	NA	NA
5	Shamsudin et al. [20], 2014	To objectively evaluate the treatment response in vitiligo using and contrasting a C-DIAS with the PGA	Computerized digital imaging analysis system (unsupervised)	56 patients above 15 (29 women, 27 men)	NA	*p* = 0.053	NA	NA
6	Zhong et al. [21], 2024	To establish a deep learning framework to enhance the diagnostic accuracy of vitiligo	ResNet networks, Swin Transformer networks	The dermoscopic image dataset utilized was sourced from the Department of Dermatology at Xijing Hospital	*n* = 4320	0.94	94.02%	93.5%
7	Kantoria et al. [22], 2020	Classification based on the deep learning–based approach to solving the problem of classifying vitiligo lesions using convolutional neural networks (CNNs) was employed	KNN, SVM, CNN, LR	Inception-V3, VGG-16, VGG-19, and SqueezeNet	*n* = 696	98% and 96.5%	NA	NA
8	Sharma et al. [23], 2023	This work has implemented a DL-based model for predicting and classifying vitiligo skin disease in healthy skin	Naïve Bayes, CNN, RF, DT	Inception V3	*n* = 1202	99%	99%	99%
9	Zhang et al. [24], 2021	To assess the performance of deep learning methods for diagnosing vitiligo by deploying CNN	CNN	DermNet DermNet NZ AtlasDerm DermIS SD-260 Kaggle DanDerm	*n* = 2876	99%	NA	NA
10	Guo et al. [25], 2022	To establish and validate a deep learning–based hybrid AI model for the objective morphometric and colorimetric assessment of vitiligo lesions	CNN	YOLO v3, UNet++	*n* = 3982	0.79 on main dataset, 0.69 on additional test set	92.91% and 72.41%	NA
11	Dixit and Sharma [26], 2024	Introduce a DL-based model for predicting and categorizing vitiligo in healthy skin	Naive Bayes, CNN, RF, DT	Inception V3	NA	99%	99%	NA

Conference								
1	Anthal, Upadhyay, and Gupta [27], 2017	To apply the LVQ NN to detect disease in the vitiligo image in affected versus unaffected areas	LVQ neural network (supervised)	Internet	NA	Ac = 92.22%	NA	NA
2	Das Gupta et al. [28], 2015	This article presents a symmetric KL divergence based on a multilevel segment agglomerative clustering system in vitiligo frames	KL divergence–based agglomerative clustering (unsupervised)	35 patients (15 female, 20 male)	*n* = 52	*p* value < 0.05	NA	NA
3	Low, Huang, and Raina [29], 2020	To introduce a (CNN) that quickly and robustly performs such segmentations without manual intervention	U-Net-based CNN (supervised)	Patients (no. of patients NA)	*n* = 308	JI = 73.6%	NA	NA
4	Bian et al. [30], 2019	To present a new, weakly supervised system for high-quality vitiligo segment areas, a fundamental task for vitiligo evaluation	CNN with saliency propagation (supervised)	Vit2019 (80% patients, 20% Internet, e.g., Google Images DERM 101)	*n* = 2000	NA	NA	NA
5	Gupta et al. [31], 2018	A new approach to diagnose different skin events (vitiligo) due to diabetes	CNN with SVM (supervised)	NA	*n* = 996	Ac = 91%	NA	NA
6	Mehmood, Khan, and Rashid [32], 2022	This article provides a method for segmenting skin lesions using a clustering algorithm	*k*-means clustering	NA	*n* = 500	NA	NA	NA

Abbreviations: Ac, accuracy; ANN, artificial neural network; C-DIAS, computerized digital imaging analysis system; CNN, convolutional neural network; DT, decision tree; FCM, fuzzy *C*-mean; ICA, independent component analysis; JI, Jaccard index; LVQ, learning vector quantization; MLP, multilayer perceptron; NA, not available; NN, neural network; PGA, physician's global assessment; RF, random forest.

## Data Availability

The details of the data and material are available within this article.
